# Engineering siRNA-loaded and RGDfC-targeted selenium nanoparticles for highly efficient silencing of DCBLD2 gene for colorectal cancer treatment

**DOI:** 10.1186/s11671-023-03870-0

**Published:** 2023-07-21

**Authors:** Hongli Huang, Hanqing Chen, Diwen Shou, Ying Quan, Jiemin Cheng, Huiting Chen, Gang Ning, Yongqiang Li, Yu Xia, Yongjian Zhou

**Affiliations:** 1 Department of Gastroenterology and Hepatology, Guangzhou First People’s Hospital, School of Medicine, South China University of Technology, Guangzhou, 510180 China; 2 Guangzhou Key Laboratory of Digestive Diseases, Guangzhou Digestive Disease Center, Guangzhou First People’s Hospital, Guangzhou, 510180 China

**Keywords:** Selenium nanoparticles, Small interfering RNA, Endocytosis, Colorectal cancer, Combination therapy

## Abstract

Effective and safe delivery of small interfering RNA (siRNA) by nanomaterials to cancer cells is one of the main challenges in cancer treatment. In this study, we constructed the selenium nanoparticles conjugated with RGDfC (one tumor-targeted polypeptide) to prepare a biocompatible gene vector (RGDfC-SeNPs) and then loaded with siDCBLD2 to synthesize the RGDfC-Se@siDCBLD2 for colorectal cancer (CRC) therapy. As expected, RGDfC-SeNPs could enhance the cellular uptake of siDCBLD2 in human HCT-116 colon cancer cells by targeting polypeptide RGDfC on the surface of colon cancer cells. RGDfC-Se@siDCBLD2 could be effectively internalized by HCT-116 cells mainly through a clathrin-related endocytosis pathway. In addition, RGDfC-Se@siDCBLD2 exhibited high siRNA release efficiency in an acidic tumor environment. Moreover, RGDfC-Se@siDCBLD2 could inhibit the proliferation and induce apoptosis in HCT-116 cells by special silencing gene DCBLD2 expression. RGDfC-Se@siDCBLD2 could be specifically accumulated to the tumor sites and exhibited significantly anti-CRC efficacy on HCT-116 tumor-bearing mice without obvious side effects. Taken together, these results suggest that selenium nanoparticles can be used as an effective gene vector with good biocompatibility, and RGDfC-Se@siDCBLD2 provides a promising strategy for combining tumor-target and siRNA delivery in treating CRC.

**Supplementary Information:**

The online version contains supplementary material available at 10.1186/s11671-023-03870-0.

## Introduction

Colorectal cancer (CRC) takes the third and second places in terms of morbidity and cancer-related death worldwide, indicating that CRC is a prevalent malignancy globally with an estimated burden increase to over 2.2 million new diagnosed cases and 1.1 million fatalities in 2030 in the world [1, 2]. In China, the rate of morbidity and mortality of patients with CRC is higher than the global average due to the growth and aging of the population, as well as the changes in at-risk behaviors and lifestyles [3]. With the recent development of therapeutic strategies, the survival rate for CRC patients has been significantly improved [4]. Unfortunately, the mainstay of the treatment for CRC patients is surgery along with adjuvant chemotherapy, and patients with CRC still suffer from drug resistance, low efficacy and high toxicity [5]. The dilemma suggests that it is an urgent need to develop a novel and promising therapeutic strategy in the treatment of CRC [6].

Small interfering RNA (siRNA), a short double-stranded RNA with 21–23 nucleotides in length and 2 nm in diameter, has high specificity for sequences to the therapeutically relevant genes and exhibited a high ability to decrease target gene levels in a variety of cancer models [7–10]. RNA interference (RNAi)-based therapeutic technology has emerged as a promising strategy in patients with CRC [11]. However, it is difficult to deliver the therapeutic siRNA into the targeted cancer cells and tissues without the assistance of appropriate carriers [12, 13]. Generally, the delivery of siRNA for gene therapy was implemented with viral and non-viral carriers [14, 15]. Although viral carriers are effective in achieving gene silencing, the risk of insertional mutagenesis and safety concerns over the immunogenicity of the viral vectors have limited their further clinical application [16]. In contrast, non-viral vectors are considered to have greater application potential because of their safety property [17]. Therefore, it is imperative to develop a safe and effective non-viral delivery system for siRNA in cancer treatment [18].

Effective delivery of siRNA to tumor lesions is currently the biggest challenge in the clinical application of siRNA-based drugs for the treatment of cancers. Recently, nanoparticles (NPs) have been successfully used as novel drug/siRNA delivery systems in the treatment of cancer owing to their unique properties [19, 20]. As anti-tumor drug carrier, NPs have inherent advantages, mainly reflected in two aspects: first, NPs have high permeability long retention effect (EPR), which is easier to penetrate into tumor tissues than normal tissues, and the retention time is longer; second, NPs can flexibly change the size and volume to better load and deliver anti-tumor drugs [21–23]. Recently, using nanocarriers to selectively deliver siRNAs to silence cancer-related genes for cancer therapy remains a challenge. Polymeric-based nanoparticles could be specifically targeted to tumors with increased therapeutic potential in mammary carcinoma [24]. Gold nanoparticles as gene delivery vehicles could significantly enhance gene silencing in tumor tissues [25]. Moreover, emerging evidence has confirmed that the enhanced accumulation of nanoparticles in solid tumors via systemic administration promotes the ability to target tumor cells and the cell delivery of siRNA [26]. Therefore, developing and designing novel functionalized nanoparticles as non-viral vectors for siRNA delivery has become a promising strategy for CRC treatment [27].

**Scheme 1.**
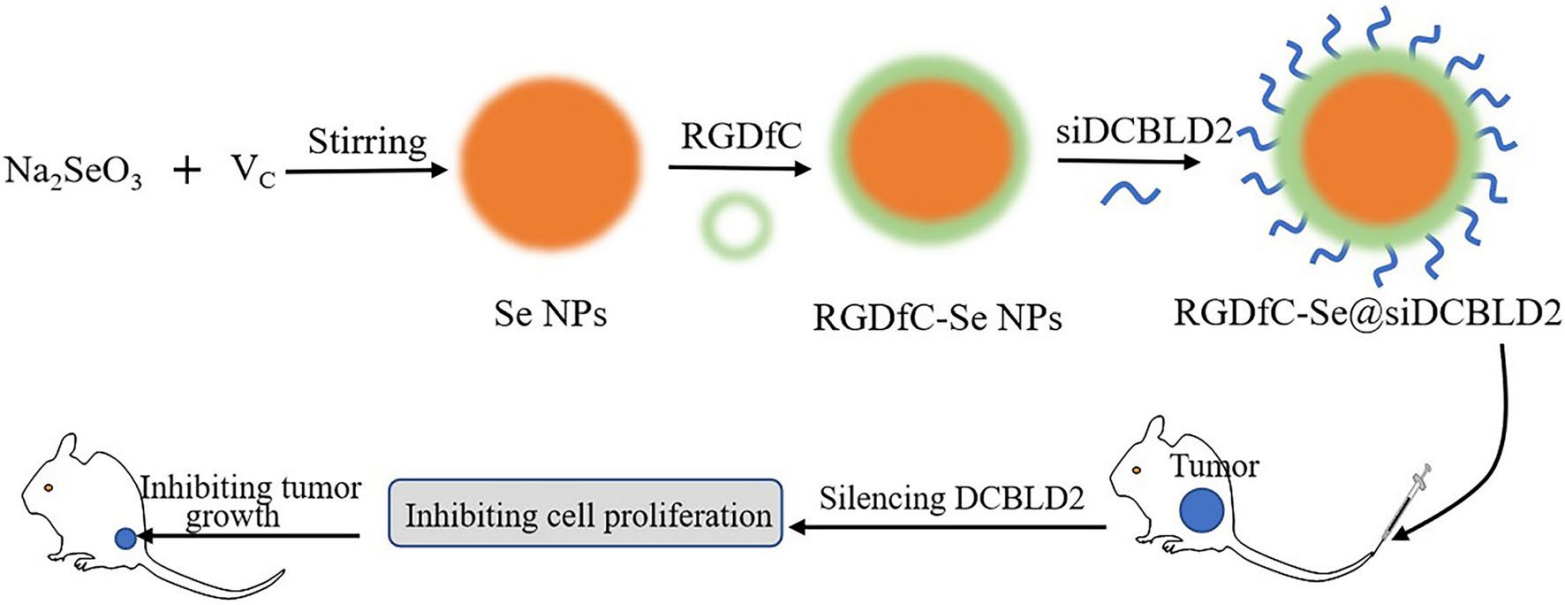
Overall schematic diagram of the formation of RGDfC-Se@siDCBLD2 and its antitumor activity

Selenium nanoparticles (Se NPs) have garnered increasing attention as potential drug/siRNA vectors and therapeutic agents for cancer treatment [28, 29]. Selenium (Se) as human trace element plays an important role for preventing cancer, and the metabolites of Se NPs can be partially absorbed by human body, which ensures a proper function of the immune system [30]. Se NPs have exhibited excellent antioxidant activity and biocompatibility due to their excellent biological activity [31]. RGDfC can effectively bind to siRNA and protects siRNA from rapid degradation by plasma nuclease [32]. Moreover, RGDfC can specifically target tumor endothelium due to the specific interaction of RGDfC and its receptors α_v_β_3_/α_v_β_5_ integrin, which are overexpressed on various cancer cells, including CRC. RGDfC ensures better enrichment of siRNA-loaded Se NPs in the periphery of tumor cells by specific interaction between RGDfC and its receptors α_v_β_3_/α_v_β_5_ integrin, and Se NPs makes more siRNA uptake by tumor cells through EPR effect [33]. In addition, silencing DCBLD2 could inhibit the cancer cells differentiation and migration and induce it apoptosis [34, 35]. Thus, we need to further clarify whether silencing DCBLD2 by siRNA delivery platform exhibit good antitumor efficacy in tumor-bearing mice model. In order to enhance the negatively charged siRNA loading efficacy, Se NPs were conjoined with the positively charged polypeptide RGDfC on the surface (RGDfC-SeNPs). Then an NP-based siRNA delivery platform (RGDfC-Se@siDCBLD2) was formed by encapsulation of DCBLD2 siRNA (siDCBLD2). RGDfC-Se@siDCBLD2 exhibited significantly antitumor efficacy in HCT-116 colon cancer cells and CRC mouse model by increasing the oncogenic gene silencing ability (Scheme 1). These findings altogether indicated that Se NPs could be used as the potential siRNA-based drug delivery platform, and RGDfC-Se@siDCBLD2 showed a novel therapeutic strategy for CRC treatment.

## Materials and methods

### Materials

Sodium selenite (Na_2_SeO_3_) and ascorbic acid (vitamin C) were purchased from Guangzhou Chemical Reagent Factory (Guangzhou, PR China). Fetal bovine serum (FBS) and phosphate buffer saline (PBS) was purchased from Gibco (Life Technologies AG, Switzerland). Dulbecco’s modified Eagle’s medium (DMEM) was purchased from Invitrogen. 3-(4,5-Dimethylthiazol-2-yl)-2,5-diphenyltetrazolium bromide (MTT) was purchased from Beyotime. Annexin V-FITC Apoptosis Kit was purchased from Sinopharm (Shanghai, PR China). 4,6-Diamidino-2-phenylindole (DAPI) was purchased from Sigma (St. Louis, MO). The cyclic polypeptide Arg-GlyAsp-D-Phe-Cys (RGDfC) was purchased from China Polypeptides Co., Ltd. The siDCBLD2, siNC (Negative control, scramble sequence), FAM-labeled siDCBLD2, and cy5.5-labeled siDCBLD2 were obtained from RiboBio (Guangzhou, PR China), and the siDCBLD2 and siNC were as follows: 5′-GGCCAAAUCAGUGUUGUAAUU-3′ and 5′-UUCUCCGAACGUGUCACGUTT-3′. All the antibodies were purchased from CST (Massachusetts, US). All of the chemicals were analytical reagent grade and used according to previously published article [8].

### Synthesis of RGDfC-Se NPs and RGDfC-Se@siDCBLD2

The synthesis of RGDfC-Se NPs and RGDfC-Se@siDCBLD2 were referred to previously published article [8]. The RGDfC-modified selenium nanoparticles (RGDfC-Se NPs) were synthesized as follows: 5 mL ascorbic acid (4 mM) was added slowly to 5 mL sodium selenite (1 mM), and the mixed solution was stirred for 4 h to obtain selenium nanoparticles (Se NPs). Then, 1 mg of RGDfC dissolved in deionized water was added to the Se NPs solution. Next, the solution was stirred for 2 h to prepare the RGDfC-modified selenium nanoparticles (RGDfC-Se NPs). The final conjugation ratio between Se NPs and RGDfC was about 5:1. The mixed solution was dialyzed (3.5 kDa) to remove the redundant ascorbic acid, sodium selenite, and RGDfC. The RGDfC-Se@siDCBLD2 were prepared as follows: the RGDfC-Se NPs dispersed in DNase/RNase-free water were vortexed with a siDCBLD2 solution for 30 min to obtain RGDfC-Se@siDCBLD2. The synthesis of RGDfC-Se@siNC is similar to the above. To determine the loading capacity of RGDfC-Se NPs on siDCBLD2, the calibration curve against FAM- siDCBLD2 was established. Subsequently, we measured the relative fluorescence intensity of FAM-siDCBLD2 loaded on RGDfC-Se NPs. The test was carried out by a multi-mode plate reader (PerkinElmer, USA). The excitation wavelength of FAM-siDCBLD2 is 465 nm, and the emission wavelength is 520 nm.

The loading efficiency of siDCBLD2 was calculated using the following formula according to previously published article [14]: loading efficiency (%) = (weight of loaded siDCBLD2/weight of siDCBLD2 in feed) × 100%. The loading content (%) = (weight of loaded of siDCBLD2/weight of RGDfC-Se@ siDCBLD2) × 100%. The loading efficiency and loading content of siDCBLD2 onto RGDfC-Se NPs were about 58.3% and 6.2 wt%, respectively.

### Characterization of the NPs

The morphology of the NPs was recorded by Hitachi transmission electron microscope (TEM). The elemental composition of the NPs was detected by HRTEM-EDX analysis on an EX-250 system (Horiba). Size distributions and zeta potentials of the NPs were measured by a Nanoparticle analyzer (HORIBA) as previously reported [14].

### A agarose gel retardation assay

The concentration of siDCBLD2 was fixed at 1 μM, and RGDfC-Se@siDCBLD2 with various RGDfC-Se/siDCBLD2 weight ratios were prepared. An agarose gel retardation assay was performed to determine the stability of RGDfC-Se@siDCBLD2 with various RGDfC-Se/siDCBLD2 weight ratios. RGDfC-Se@siDCBLD2 were subjected to gel electrophoresis in 1% agarose gel with 0.5 mg/ml ethidium bromide (EB) at 120 mV for 15 min. The UV gel image system was used to visualize the gel according to previously published article [8].

### Cell culture

Human colon cancer cells (HCT-116) and human normal colon cells (NCM460) were purchased from the American Type Culture Collection (ATCC). Both cells were incubated in Dulbecco's modified Eagle’s medium (DMEM) containing 10% FBS and 1% penicillin/streptomycin at 37 °C in a humidified atmosphere with 5% CO_2_ according to the method in previously published article [14].

### Cellular uptake of NPs

HCT-116 cells were plated into 24-well plates overnight at 37 °C, and then the cells were incubated with RGDfC-Se@FAM-siDCBLD2 at a siDCBLD2 concentration of 100 nM for 1, 2, or 4 h. In the experimental section of cell pretreatment with RGDfC, the cells were pre-treated with 1 μg/mL of RGDfC for 4 h and then incubated with RGDfC-Se@FAM-siDCBLD2 at a siDCBLD2 concentration of 100 nM for 4 h. Subsequently, the cells were washed with cold phosphate-buffered saline (PBS) for three times, fixed in 4.0% paraformaldehyde for 20 min at room temperature, and then incubated with 2 μg/mL DAPI for 10 min. The result was observed using confocal microscopy (Zeiss, Oberkochen, Germany) according to the method in previously published article [17].

### The uptake pathways of RGDfC-Se@siDCBLD2

The cellular uptake pathways of NPs were investigated using HCT-116 cells, which were incubated at 4 °C for 0.5 h or with the following endocytosis-related inhibitors, respectively: NaN_3_ (sodium azide, 3 mg/mL) + 50 mM 2-deoxy-D-glucose (DOG), nystatin (5 mg/mL), chlorpromazine (5 mg/mL) and amiloride (10 mg/mL) in serum-free medium at 37 °C for 30 min. The uptake of HCT-116 cells was quantitatively analyzed by flow cytometry after treatment (BD, FACS Aria) as previously reported [17].

### SiDCBLD2 release efficiency

To determine the release efficiency of siDCBLD2 from the surface of NPs, RGDfC-Se/siDCBLD2 with a weight ratio of 8/1 was incubated in 10 mM HEPES buffer at pH 5.4 or 7.4. The samples were taken out at different time points and then centrifuged at 4000 rpm for 8 min to harvest the free siDCBLD2. The concentration of siDCBLD2 was measured by a SpectraMax QuickDrop as previously reported [29].

### Western blot

The HCT-116 cells were co-cultured with RGDfC-Se@siDCBLD2 or RGDfC-Se@siNC for 24 h, and then the cells were washed with PBS. The proteins were extracted using RIPA lysis buffer, and protein concentrations were determined by a BCA Protein Assay kit (Beyotime, Nantong, China). The collected proteins were further analyzed as previously reported [36].

### Cytotoxicity assay

HCT-116 cells or NCM460 cells were seeded at a density of 5 × 10^4^ cells per well in 96-well culture clusters to incubate for 24 h at 37 °C. In the in vitro anti-cancer experiments, the HCT-116 cells were incubated with RGDfC-Se@siDCBLD2 or RGDfC-Se@siNC in various siRNA equivalent concentrations for another 48 h at 37 °C. To investigate the biocompatibility of RGDfC-Se@ siDCBLD2, the human normal colon cells NCM460 cells were treated with RGDfC-Se@siDCBLD2 in various siDCBLD2 concentrations for 48 h at 37 °C. After that, the cell viability was tested using the MTT assay as previously reported [37].

### Cell apoptosis analysis

Cell apoptosis analysis was carried out by FACS flow cytometer according to previously published article [8]. HCT-116 cells were seeded in 12-well plates until about 60% confluence. The cells were treated with RGDfC-Se@siDCBLD2 or RGDfC-SeNPs@siNC (siRNA equivalent concentrations 100 nM) for 48 h at 37 °C, and the untreated cells were set as a control group. After treatment, the cells were harvested and fixed with 70% ethanol at 4 °C for 6 h. Then the fixed cells were stained with propidium iodide for 15 min. FACS flow cytometer was used to analyze the cell cycle distribution and apoptosis. The sub-G1 peak was defined as the apoptotic peak. The result was analyzed by the FlowJo software (Treestar, USA) as previously reported [29].

### Trans-well assay

The cell migration was investigated by trans-well assay (8 mm pore size, BD FalconTM, USA). The HCT-116 cells in the apical side chamber were incubated with RGDfC-Se@siDCBLD2 or RGDfC-SeNPs@siNC at siRNA equivalent concentrations of 100 nM for 48 h at 37 °C, and the untreated cells were set as a control group. The migrated HCT-116 cells on the bottom closet were fixed by 4% paraformaldehyde and stained with 0.1% crystal violetin. Each step was followed by rinsing gently with cold PBS three times. The images of cells on the bottom were obtained by an inverted microscope (CK2, Olympus, Japan) as previously reported [8].

### In vivo biodistribution

The animal protocol was approved by the Animal Experimentation Ethics Committee of Guangdong Medical Laboratory Animal Center in accordance with the guidelines and regulations of Animal Experimentation Ethics Committee of Guangdong Medical Laboratory Animal Center. The maximal tumour size permitted by the ethics committee is 1.5 cubic millimeters, and all tumors in this study were not exceeded the maximal tumour size. 6-week-old BALB/c nude mice were injected with HCT-116 cells (1 × 10^7^) subcutaneously. After the tumor grew approximately 200 mm^3^, RGDfC-Se@cy5.5-siDCBLD2 were injected into mice via a tail vein. At 3 h post-injection, the anesthetized mice were placed in the chamber, and the fluorescence images were detected by an IVIS imaging system (Xenogen, USA) as previously reported [8].

### Xenograft mouse model

BALB/c nude mice (6-week ages) were injected with HCT-116 cells (1 × 10^7^) subcutaneously to build the tumor mouse model. When the tumor grew to about 100 mm^3^, the mice (*n* = 6) were intravenously administered with RGDfC-Se@siDCBLD2 (1.0 mg/kg), RGDfC-SeNPs@siNC (1.0 mg/kg), and an equal volume of saline once every other day, respectively. The weight of the mice was recorded every 3 days. After 16 days, the mice were sacrificed and the tumors, heart, liver, spleen, lung, and kidney were harvested for analysis.

### Histology and immunohistochemistry

The tumor tissues and the main organs tissues, including the heart, liver, spleen, lung, and kidney, were fixed with 3.7% paraformaldehyde, embedded in paraffin, and sectioned. The tumors and main organs sections were stained with hematoxylin and eosin (H&E) for histological analysis as previously reported. Tumor cell growth (Ki67)-, apoptosis (caspase-3 and TUNEL)- or angiogenesis (CD34)-related proteins in tumor tissues were further measured by immunohistochemistry according to the manufacturer's protocols. The images of sections were obtained by using a digital microscope (Leica DMi8).

### Statistical analysis

All experiments were repeated three times, and the analysis of variance was carried out by Student’s *t* test. Statistical significance was indicated by *p < 0.05, **p < 0.01, or ***p < 0.001. The differences among different groups were verified by one-way ANOVA multiple comparisons.

## Results and discussion

### Synthesis and physicochemical characterization of RGDFC-SeNPs and RGDfC-Se@siDCBLD2

**Fig. 1:**
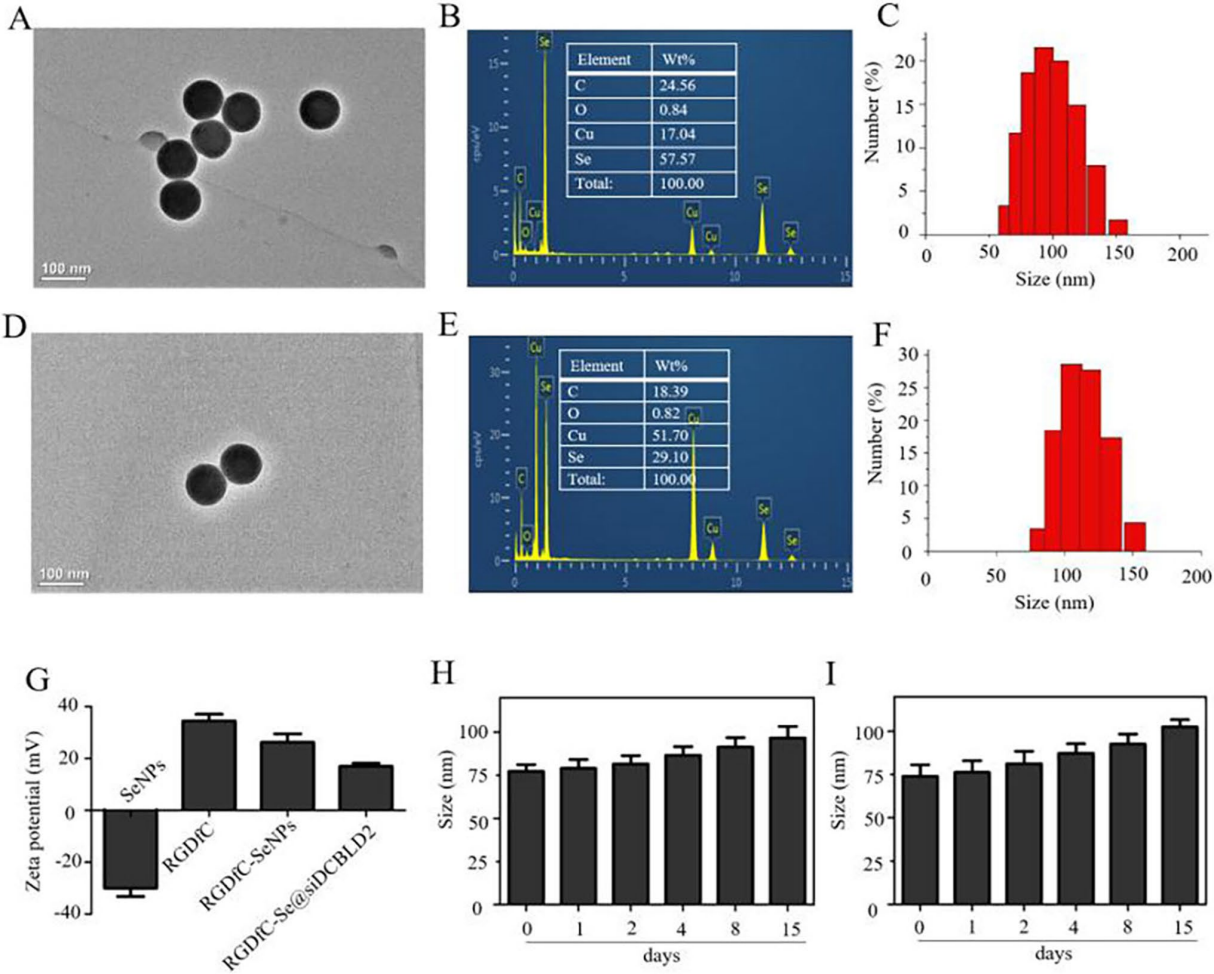
Physicochemical characterization of RGDFC-SeNPs and RGDfC-Se@siDCBLD2. Representative TEM images of RGDFC-SeNPs (**a**) and RGDfC-Se@siDCBLD2 (**d**). TEM in combination with energy-dispersive X-ray spectroscopy (EDX) analysis of the main elemental compositions in RGDfC-SeNPs (**b**) and RGDfC-Se@siDCBLD2 (**e**). The size distribution of RGDfC-SeNPs (**c**) and RGDfC-Se@siDCBLD2 (**f**) was determined by TEM analysis. **g** Zeta potentials of SeNPs, RGDfC, RGDfC-SeNPs and RGDfC-Se@siDCBLD2. Dynamic light scattering (DLS) analysis of the stability of RGDfC-Se@siDCBLD2 in MilliQ water (**h**) and phosphate-buffered saline (PBS) (**i**) during 15 days

Herein, selenium nanoparticles (Se NPs), as gene delivery vectors, were prepared by adding ascorbic acid to a selenite aqueous solution. To enhance the tumor-targeting ability of Se NPs, we conjoined the positively charged polypeptide RGDfC on the surface of Se NPs by the electrostatic interactions to prepare RGDfC-Se NPs. Subsequently, siRNA targeting to DCBLD2 (siDCBLD2) was modified with the RGDfC through the electrostatic interactions to obtain the RGDfC-Se@siDCBLD2. The loading efficiency of siDCBLD2 (50.97 ± 7.47)% was determined by UV spectrophotometer. The detailed physicochemical characteristics of the RGDFC-SeNPs and RGDfC-Se@siDCBLD2 are listed and shown in Fig. 1 and Fig. S1–S2. Transmission electron microscopy (TEM) images showed that RGDfC-SeNPs (Fig. 1a) and RGDfC-Se@siDCBLD2 (Fig. 1d) exhibited spherical morphology and smooth surface with a narrow diameter of about 85 nm. TEM in combination with energy-dispersive X-ray spectroscopy (EDX) indicated that the main elemental compositions in RGDfC-SeNPs and RGDfC-Se@siDCBLD2 included selenium (Se), carbon (C), and oxygen (O). The elemental weight percentage of RGDfC-SeNPs and RGDfC-Se@siDCBLD2 are presented in Fig. 1b, e. The dynamic light scattering (DLS) particle size and *polymer dispersity index* (PDI) value of RGDFC-SeNPs and RGDfC-Se@siDCBLD2 were 86.7 nm, 0.095 and 106.6 nm, 0.143 (Fig. 1c, f), respectively. Meanwhile, well-defined characterized peaks of SeNPs were prominently apparent at 1635 cm^−1^, and the peaks of amide bands attributed to RGDfC at 1525 were obtained in RGDFC-SeNPs and RGDfC-Se@siDCBLD2 characterized by the Fourier transform infrared (FTIR) spectroscopy (Fig. S1), which indicated the successful synthesis of RGDFC-SeNPs and RGDfC-Se@siDCBLD2. The zeta potential of SeNPs was about − 29 mV, and was changed to + 27.8 mV and + 14.9 mV for RGDFC-SeNPs and RGDfC-Se@siDCBLD2, respectively (Fig. 1g). The DLS of RGDfC-Se@siDCBLD2 in MilliQ water and PBS were not changed within 15 days (Fig. 1h, i), indicating the biocompatibility and stability of RGDfC-Se@siDCBLD2. The siDCBLD2 loading ability of RGDfC-SeNPs was furtherly evaluated by agarose gel electrophoresis [34]. When the weight ratio of RGDfC-SeNPs/siDCBLD2 increased to 8:1 (Fig. S2), complete retardation of siDCBLD2 migration could be observed, indicating that RGDfC-SeNPs was able to protect siDCBLD2 from degradation during electrophoresis process. No obvious precipitate was observed in the mixed solutions including the serum protein containing media and RGDfC-Se@DCBLD2, indicating good stability siDCBLD2 in RGDfC-Se@DCBLD2 (Fig. S3).

### Cellular uptake of RGDfC-Se@siDCBLD2 in HCT-116 cells

**Fig. 2:**
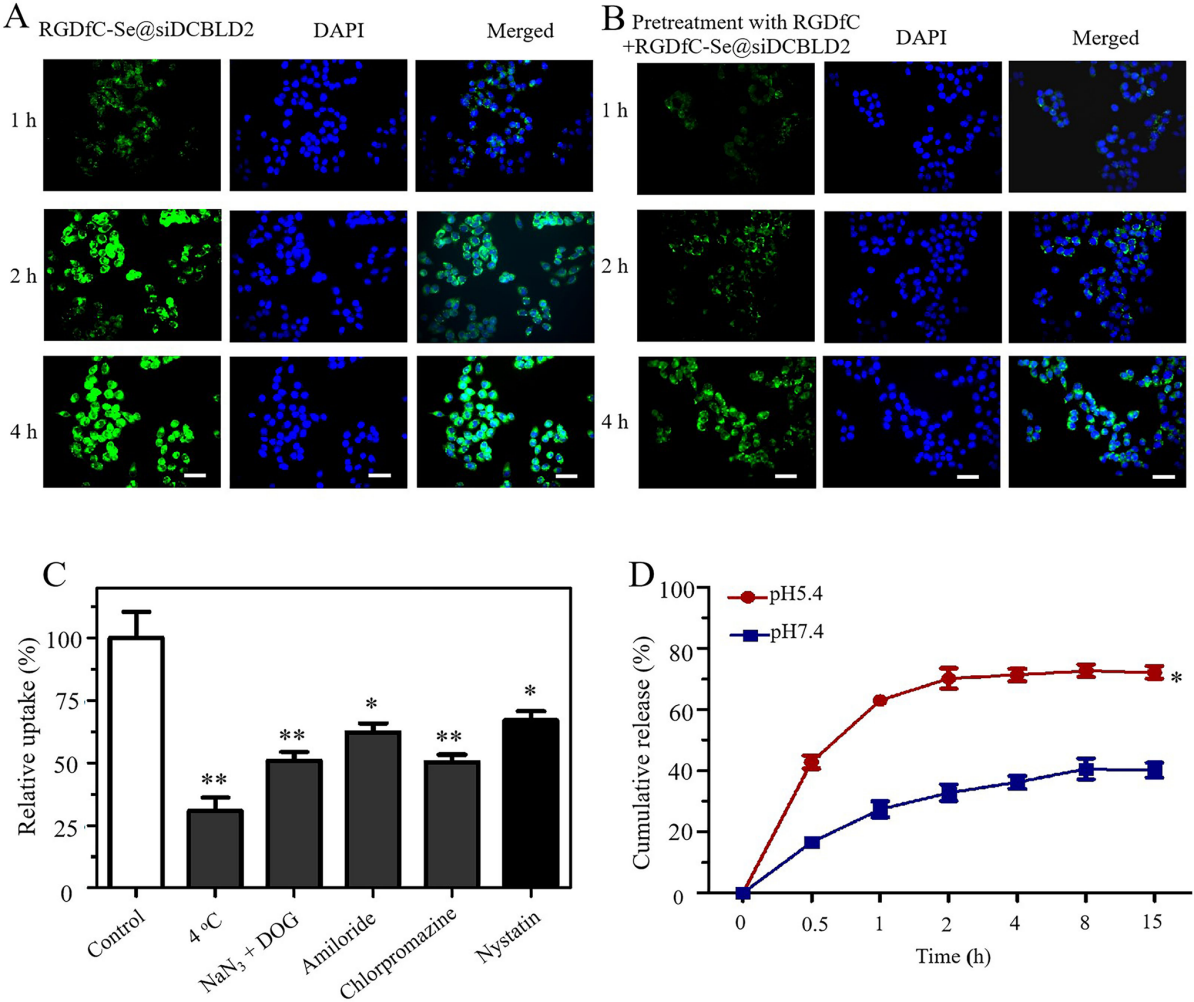
Cellular uptake of NPs. **a** Cellular uptake of RGDfC-Se@siDCBLD2 in the HCT-116 cells was observed by laser scanning confocal microscopy (LSCM). Scale bar, 40 μm. Green fluorescence indicates RGDfC-Se@siDCBLD2, and blue fluorescence indicates the nucleus. **b** Cellular uptake of RGDfC-Se@siDCBLD2 in the HCT-116 cells pretreated with RGDfC for 4 h was observed under a fluorescence microscope. Scale bar, 40 μm. **c** Investigation of internalization pathway of RGDfC-Se@siDCBLD2 in HCT-116 cells at 4 °C incubation or the presence of different endocytosis inhibitors. *p < 0.05 and **p < 0.01. **d** The percentage of cumulative siDCBLD2 release from RGDfC-Se@siDCBLD2 under incubation conditions of pH 5.4 or 7.4. *p < 0.05

The qualitative cellular uptake of polypeptide RGDfC-modified SeNPs in HCT-116 cells (human colon cancer cells) was measured by laser scanning confocal microscopy (LSCM). As shown in Fig. 2a, HCT-116 cells were incubated with RGDfC-Se@siDCBLD2 for 1, 2, and 4 h, and then visualized the RGDfC-Se@siDCBLD2 in the cells and cell nuclei by fluorescence intensity of FAM-siDCBLD2 (green) and DAPI (blue), respectively. The green fluorescence was observed in the cytoplasm of the HCT-116 cells after 1 h of incubation, and the fluorescence intensity of RGDfC-Se@FAM-siDCBLD2 inside the HCT-116 cells was progressively enhanced with increasing incubation time from 2 to 4 h. This result indicated that RGDfC-Se@siDCBLD2 had a high cellular uptake efficiency, and the process of cellular uptake of RGDfC-Se@siDCBLD2 depended on the incubation time. To further investigate the effect of polypeptide RGDfC on the efficiency of cellular uptake of RGDfC-Se@siDCBLD2, we pretreated the HCT-116 cells with RGDfC for 4 h before treatment with RGDfC-Se@FAM-siDCBLD2, and the cells were then co-incubated for 4 h. As expected, the cellular uptake of RGDfC-Se@siDCBLD2 in RGDfC-pretreated HCT-116 cells was significantly decreased compared with that in unpretreated cells (Fig. 2b), which can be explained that RGDfC ensures better enrichment of siRNA-loaded Se NPs in the periphery of tumor cells by specific interaction between RGDfC and its receptors α_v_β_3_/α_v_β_5_ integrin, and Se NPs makes more siRNA uptake by tumor cells through EPR effect. These results showed that the entry of RGDfC-Se@siDCBLD2 into HCT-116 cells might be mediated by specifically binding of RGDfC to the integrin receptors in cancer cells RGDfC. These results suggested that RGDfC played an important role in the targeting delivery of RGDfC-Se@siDCBLD2 in colon cancer cells.

### Internalization mechanism of RGDfC-Se@siDCBLD2 in HCT-116 cells

To further identify the internalization mechanism of RGDfC-Se@siDCBLD2 in HCT-116 cells, we investigated the effect of temperature and different endocytosis-related inhibitors on the cellular uptake of RGDfC-Se@siDCBLD2 (Fig. 2c). Compared with the untreated group, the cellular uptake of RGDfC-Se@siDCBLD2 in HCT-116 cells at 4 °C was reduced by about 69.1%, indicating that the internalization of RGDfC-Se@siDCBLD2 into HCT-116 cells was due to the energy-dependent pathway. Pretreatment with NaN_3_/DOG, a cell energy metabolism inhibitor, caused a significant reduction in the cellular uptake of RGDfC-Se@siDCBLD2 in HCT-116 cells, indicating that the endocytosis of RGDfC-Se@siDCBLD2 is an active cellular transport process dependent on energy. Amiloride, chlorpromazine, and nystatin were known as inhibitors of micropinocytosis, clathrin-associated endocytosis, and caveolae-mediated cell uptake, respectively. The cellular uptake of RGDfC-Se@siDCBLD2 in HCT-116 cells pretreated with amiloride (an inhibitor of micropinocytosis) and nystatin (an inhibitor of caveolae-mediated cell uptake) was reduced by 37.6% and 32.9%, respectively. Moreover, chlorpromazine (an inhibitor of clathrin-associated endocytosis) pretreatment significantly decreased 49.5% of the cellular uptake. HCT-116 cells treated with chlorpromazine had the least amount of uptake of RGDfC-Se@siDCBLD2, indicating that blocking the clathrin-associated endocytosis pathway severely reduced uptake of RGDfC-Se@siDCBLD2 in tumor cells. These results suggested that the clathrin-associated endocytosis pathway played a vital role in the internalization of RGDfC-Se@siDCBLD2 into HCT-116 cells.

### Release profile of siDCBLD2 from RGDfC-Se@siDCBLD2 in vitro

To determine whether the targeted gene siDCBLD2 loaded into SeNPs could be fast and stable released from RGDfC-Se@siDCBLD2 in a CRC environment, in vitro release profile was performed at pH 5.4 and 7.4. As shown in Fig. 2d, siDCBLD2 was gradually released from RGDfC-Se@siDCBLD2 within 15 h at pH 5.4 and 7.4, especially in an acidic environment. The cumulative release of siDCBLD2 in pH 5.4 presented a burst release with more than 60% in the initial 1 h and increased to 72.2% at 2 h. The release profiles of siDCBLD2 from RGDfC-Se@siDCBLD2 in pH 7.4 was only about 40.2% after 15-h incubation, indicating that siDCBLD2 was prone to be released from the RGDfC-Se@siDCBLD2 in an acidic environment. This was the case because the protonated amino acid group induced by the acidic environment increased the hydrophilicity of positively charged RGDfC and weakened the electrostatic interaction between siDCBLD2 and RGDfC-Se NPs. Meanwhile, acidolysis of the RGDfC-Se@siDCBLD2 accelerated the release of siDCBLD2 in an acidic environment [38, 39].

### DCBLD2 gene silencing efficiency of RGDfC-Se@siDCBLD2 in HCT-116 cells

**Fig. 3:**
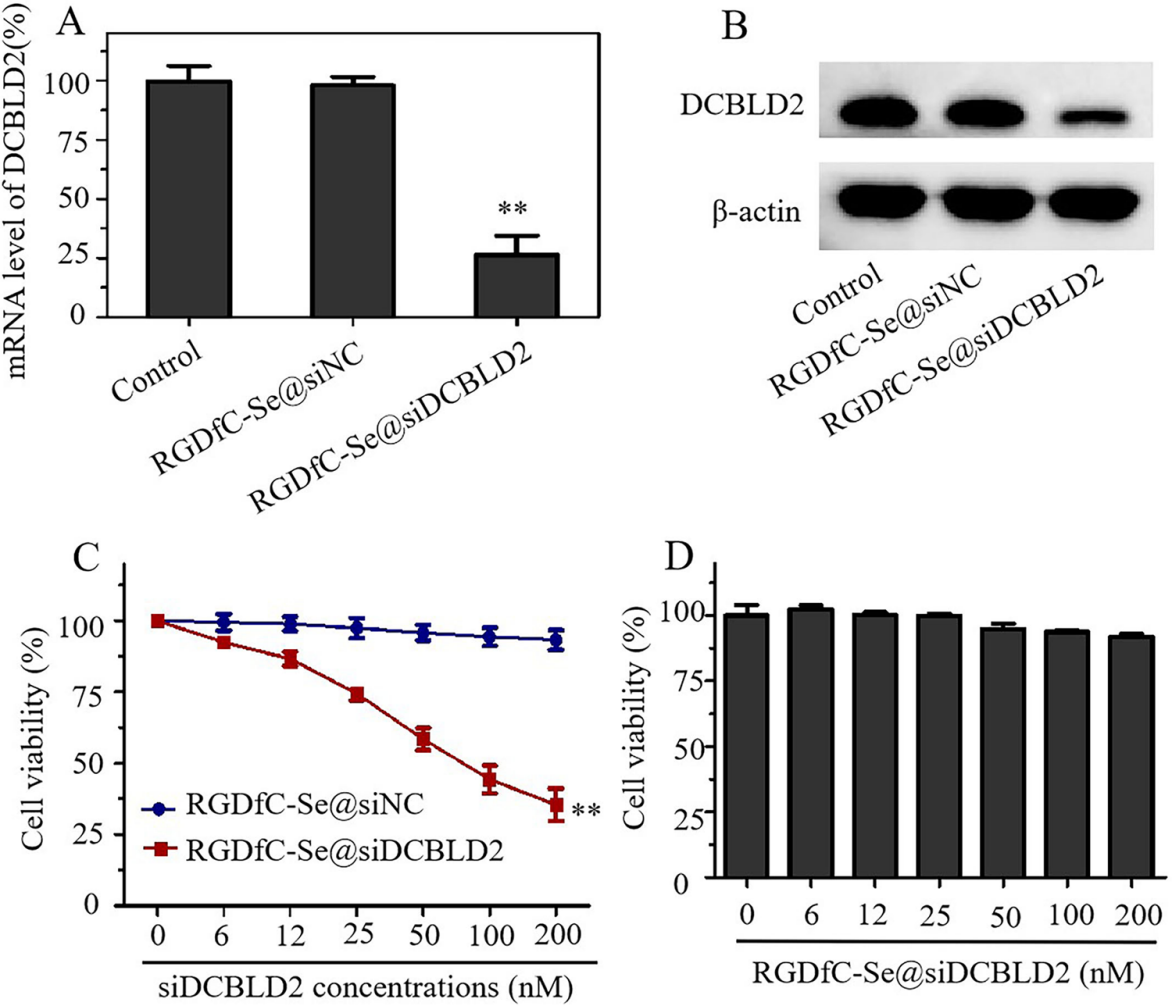
Effect of RGDfC-Se@siDCBLD2 on the DCBLD2 expression and cell viability in HCT-116 cells. **a** The mRNA level of DCBLD2 in HCT-116 cells treated with RGDfC-Se@siDCBLD2 or RGDfC-Se@siDCBLD2. **p < 0.01 versus control. **b** The protein level of DCBLD2 in HCT-116 cells treated with RGDfC-Se@siDCBLD2 or RGDfC-Se@siDCBLD2. The untreated HCT-116 cells were set as a control group. **c** In vitro cytotoxicity of RGDfC-Se@siDCBLD2 and RGDfC-Se@siNC at various siRNA concentrations on HCT-116 cells after 48 h incubation. **d** Cell viability of NCM460 cells after treatment with RGDfC-Se@siDCBLD2 for 48 h at various siDCBLD2 concentrations

Herein, we constructed the tumor-targeted functionalized selenium nanoparticles for special delivery of siDCBLD2 into the HCT-116 cells. To assess the gene silencing efficiency of RGDfC-Se@siDCBLD2, we performed quantitative polymerase chain reaction (qRT-PCR) to analyze the expression level of DCBLD2 mRNA in the HCT-116 cells after incubation with RGDfC-Se@siDCBLD2 for 24 h. Compared with the control group, a significant decrease in the expression of DCBLD2 mRNA was observed in the HCT-116 cells treated with RGDfC-Se@siDCBLD2. RGDfC-SeNPs@siNC, as a negative control group, did not affect the expression of DCBLD2 mRNA (Fig. 3a). Furthermore, the protein level of DCBLD2 was analyzed by western blot assay. HCT-116 cells treated with RGDfC-Se@siDCBLD2 for 24 h exhibited an decreased level of DCBLD2 protein compared with RGDfC-Se@siNC treatment (Fig. 3b). Therefore, their results strongly demonstrated that the RGDfC-Se@siDCBLD2 could remarkably maintain the high local concentration of siDCBLD2 and specially silence the expression of DCBLD2 in HCT-116 cells.

### Effect of RGDfC-Se@siDCBLD2 on cell viability in HCT-116 cells

Furthermore, an MTT assay was implemented to investigate the cell viability and antitumor activity of RGDfC-Se@siDCBLD2 on HCT-116 cells. Firstly, the differential expression of DCBLD2 between HCT-116 and NCM460 cells were determined by RT-qPCR and Western blot assays. The expression of DCBLD2 in HCT-116 cells was higher than that in NCM460 cells (Fig. S4, Fig. S5), indicating that DCBLD2 may develop into a good therapeutic target. Then, we want to confirm whether silencing DCBLD2 could inhibit the HCT-116 cells proliferation. As shown in Fig. 3c, RGDfC-Se@siDCBLD2 treatment at the concentration of 6 to 200 nM significant decreased the cell viability of HCT-116 cells in a dose-dependent manner, while RGDfC-Se@siNC failed to affect the cell viability of the HCT-116 cells. However, the cell viability of human normal colon cells (NCM460 cells) treated with increasing concentrations of RGDfC-Se@siDCBLD2 was comparable to untreated groups (Fig. 3d). The above results showed that RGDfC-Se@siDCBLD2 showed well biocompatibility in normal cells and exhibited excellent antitumor activity in HCT-116 cells by inhibiting cell proliferation.

### Effect of RGDfC-Se@siDCBLD2 on cell migration in HCT-116 cells

**Fig. 4:**
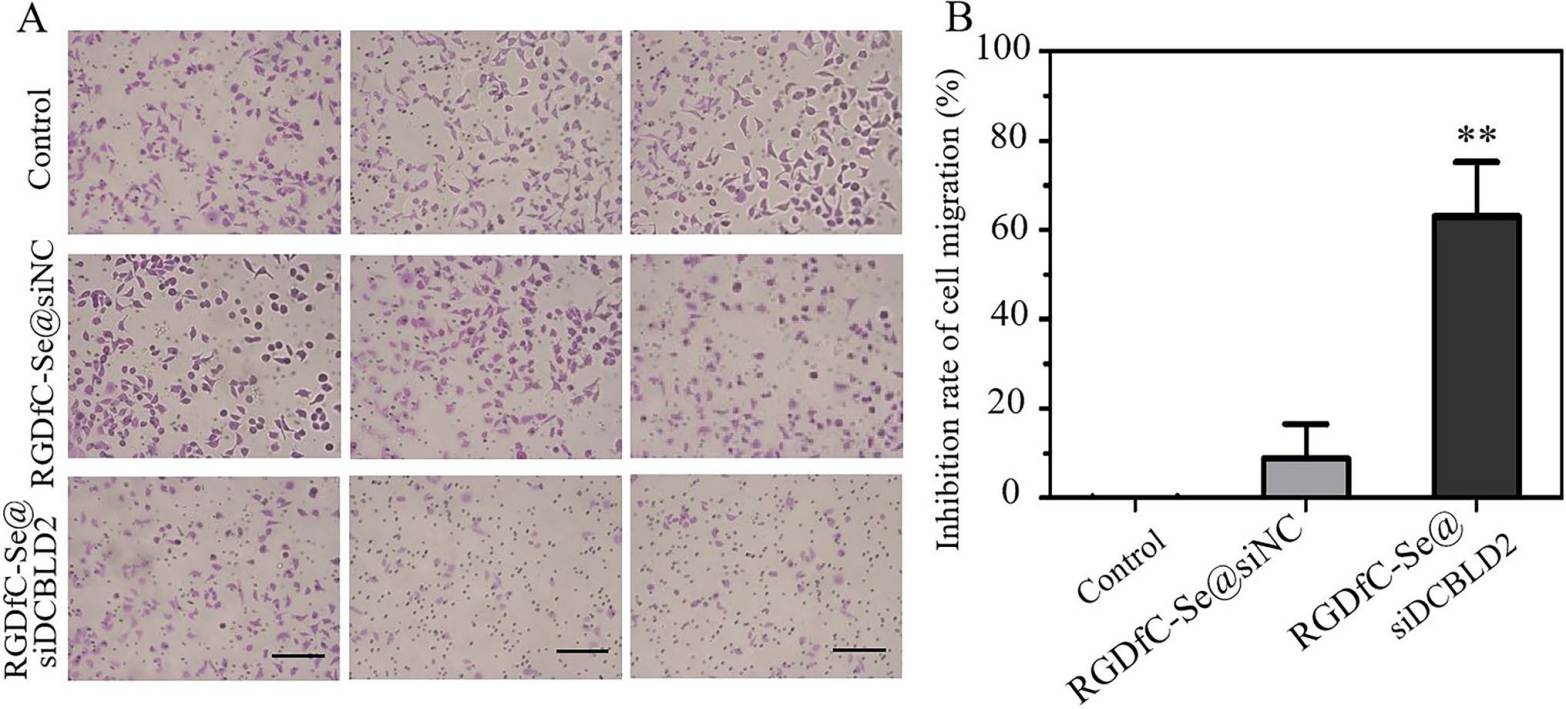
Effect of RGDfC-Se@siDCBLD2 on cell migration in HCT-116 cells. **a** The motility activity of HCT-116 cells treated with RGDfC-Se@siDCBLD2 and RGDfC-SeNPs@siNC for 24 h was determined by trans-well assay. The scale bar is 200 μm. **b** The inhibition efficiency of RGDfC-Se@siDCBLD2 and RGDfC-Se@siNC on HCT-116 cell migration in HCT-116 cells. ** indicates *p* < 0.01 versus control

We performed a trans-well assay to assess the effect of RGDfC-Se@siDCBLD2 on cell migration in HCT-116 cells (Fig. 4a). As expected, RGDfC-Se@siDCBLD2 treatment significantly reduced the number of HCT-116 cells through the trans-well chamber compared with the untreated and RGDfC-Se@siNC-treated groups. Moreover, RGDfC-Se@siDCBLD2 could weaken the migration ability of HCT-116 cells. In addition, the statistical migration inhibition rate of RGDfC-Se@siDCBLD2 was 60.03%, but RGDfC-Se@siNC was only 8.9% (Fig. 4b). The above experimental results strongly indicated that the RGDfC-Se@siDCBLD2 could inhibit the migration of tumor cells through the down-regulation of DCBLD2 expression.

### Effect of RGDfC-Se@siDCBLD2 on the distribution of cell cycle and apoptosis in HCT-116 cells

**Fig. 5:**
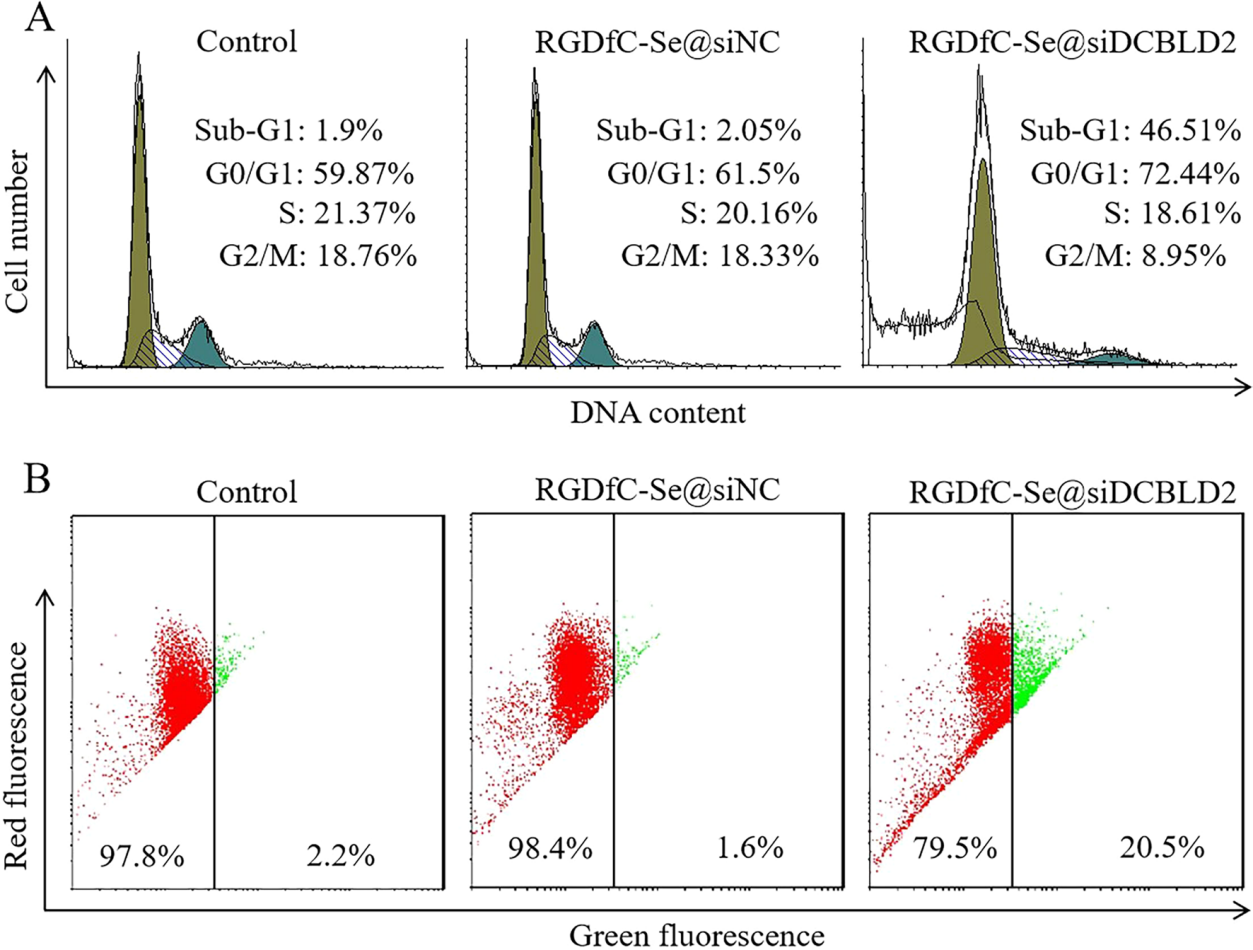
Effect of RGDfC-Se@siDCBLD2 on the distribution of cell cycle and mitochondrial membrane potential (Δψm) in HCT-116 cells. **a** Effects of RGDfC-Se@siDCBLD2 and RGDfC-SeNPs@siNC on cell cycle distribution in HCT-116 cells. **b** Effects of RGDfC-Se@siDCBLD2 and RGDfC-Se@siNC on the mitochondrial membrane potential (MMP) in HCT-116 cells. Red fluorescence indicates the cells with normal polarized mitochondrial membranes and green fluorescence indicates cells with mitochondrial dysfunction

Flow cytometry was used to analyze the cell cycle and apoptosis of HCT-116 cells after incubation with RGDfC-Se@siDCBLD2 for 24 h. As shown in Fig. 5a, the value of G0/G1 in HCT-116 cells treated with RGDfC-Se@siDCBLD2 (72.44%) was obviously increased compared with the control (59.87%) and RGDfC-Se@siNC-treated groups (61.50%). The distribution of the cell cyclein HCT-116 cells was comparable between the control and RGDfC-Se@siNC-treated groups, indicating down-regulation of DCBLD2 expression might inhibit the proliferation of colon cancer cells by arresting the cell cycle at G0/G1 phase. Additionally, the sub-G1 apoptosis peaks in the control, RGDfC-Se@siNC, and RGDfC-Se@siDCBLD2 groups were 1.9%, 2.05% and 46.51%, respectively. RGDfC-Se@siNC as negative control had little effect on apoptosis of HCT-116 cells, however, RGDfC-Se@siDCBLD2 obviously induced HCT-116 cells apoptosis, indicating RGDfC-Se@siDCBLD2 could arrest the HCT-116 cells at G0/G1 phase and further induce it apoptosis probably by silencing the DCBLD2 expression.

### Effect of RGDfC-Se@siDCBLD2 on mitochondrial membrane potential (Δψm)

Mitochondrial membrane potential (Δψm), an essential for ATP synthase to make ATP, is established by the proton transfer against their concentration gradient driven by the respiratory electron transport chains in the inner mitochondrial membrane [40]. It has been reported that the loss of Δψm could lead to the release of apoptotic factors, such as cytochrome C, and activate downstream apoptotic pathways [41]. JC-1, as a lipophilic cationic dye, was selectively capable of entering into mitochondria and reversibly changing its color from red to green when the Δψm decreased. The increase in the green fluorescence signal indicated the loss of Δψm. As shown in Fig. 5b, HCT-116 cells treated with RGDfC-Se@siDCBLD2 showed a tenfold increased green fluorescence signal compared to the control group indicating the decreased Δψm. However, there was no obvious fluorescence signal change between RGDfC-Se@siNC and control groups. These results indicated that RGDfC-Se@siDCBLD2 exhibited well antitumor in vitro by damaging the Δψm in HCT-116 cells.

### In vivo imaging of RGDfC-Se@siDCBLD2 in HCT-116 tumor xenograft model

**Fig. 6:**
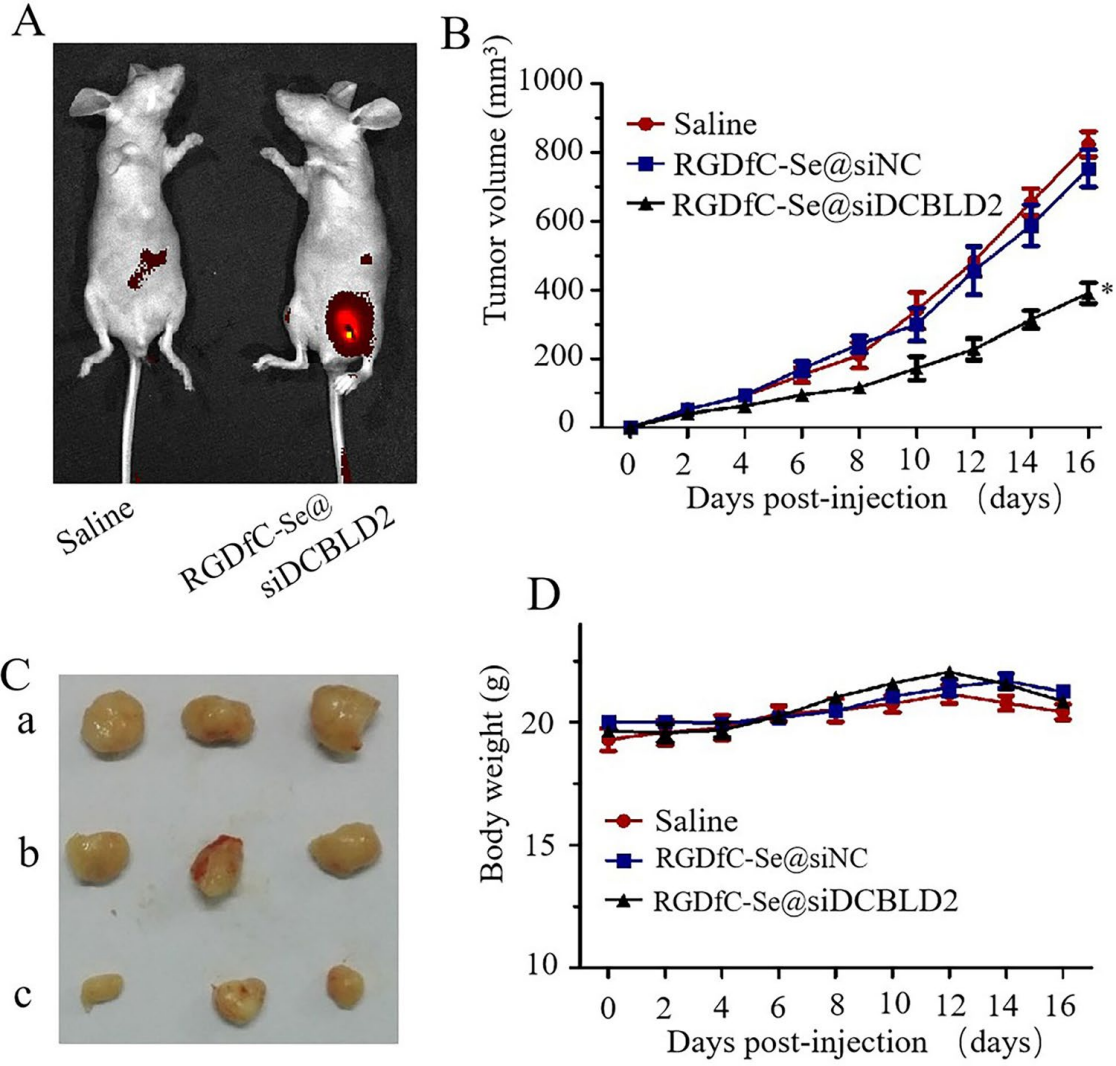
Therapeutic efficacy of RGDfC-Se@siDCBLD2 on HCT-116 tumor xenograft model. *n* = 6. **a** In vivo fluorescence images of tumor-bearing mice given RGDfC-Se@cy5.5-siDCBLD2 by intravenous injection at 3 h. Red fluorescence indicates RGDfC-Se@cy5.5-siDCBLD2. **b** Tumor volume in tumor-bearing mice after intravenous injection of saline, RGDfC-Se@siNC, or RGDfC-Se@siDCBLD2 was observed during the 16 days. **c** Digital photos of tumors in mice injected intravenously with saline, RGDfC-Se@siNC or RGDfC-Se@siDCBLD2. **d** Change in body weight of tumor-bearing mice during the treatment period

Designing and synthesizing a promising carrier to selectively deliver cancer-associated siRNA to target the tumor sites is an important strategy for cancer treatment. Herein, an in vivo imaging system was performed to detect the biodistribution of cy5.5-labeled RGDfC-Se@siDCBLD2 in the HCT-116 tumor xenograft model. As shown in Fig. 6a, the fluorescence signal of cy5.5-labeled RGDfC-Se@siDCBLD2 was significantly increased in tumor-bearing mice after intravenously injected with RGDfC-Se@siDCBLD2 for 3 h. This result suggested that RGDfC-Se@siDCBLD2 could effectively accumulate in the tumor site, which might be attributed to the specific binding of RGDfC from RGDfC-Se@siDCBLD2 and α_v_β_3_/α_v_β_5_ integrin [42].

### In vivo anti-CRC effects of RGDfC-Se@siDCBLD2 in HCT-116 tumor xenograft model

The anti-tumor efficacy of RGDfC-Se@siDCBLD2 was further assessed in the HCT-116 tumor xenograft model after intravenously injected with saline, RGDfC-Se@siNC, and RGDfC-Se@siDCBLD2, respectively. As shown in Fig. 6b, during 16 days of treatment, RGDfC showed little inhibition of tumor growth, indicating the siRNA delivery carrier itself had no effect on the tumor growth, while RGDfC-Se@siDCBLD2 began to inhibit tumor growth after 6 days of treatment, and tumors in RGDfC-Se@siDCBLD2-treatment group were suppressed by approximately 50% compared to the saline group after 16 days of treatment, indicating that RGDfC-Se@siDCBLD2 had excellent antitumor efficacy. The tumor images further demonstrated the preeminent antitumor activity of RGDfC-Se@siDCBLD2 (Fig. 6c). The DCBLD2 protein expression in RGDfC-Se@siDCBLD2 group was obviously down-regulated compared to negative control and saline groups, indicating RGDfC-Se@siDCBLD2 inhibited the tumor growth by silencing the DCBLD2 (Fig. S6). In addition, the weight of mice was recorded every other day up to 16 days. No significant weight loss was observed during the treatment, indicating that RGDfC-Se@siDCBLD2 had nearly no side-effect in tumor-bearing mice (Fig. 6d).

**Fig. 7:**
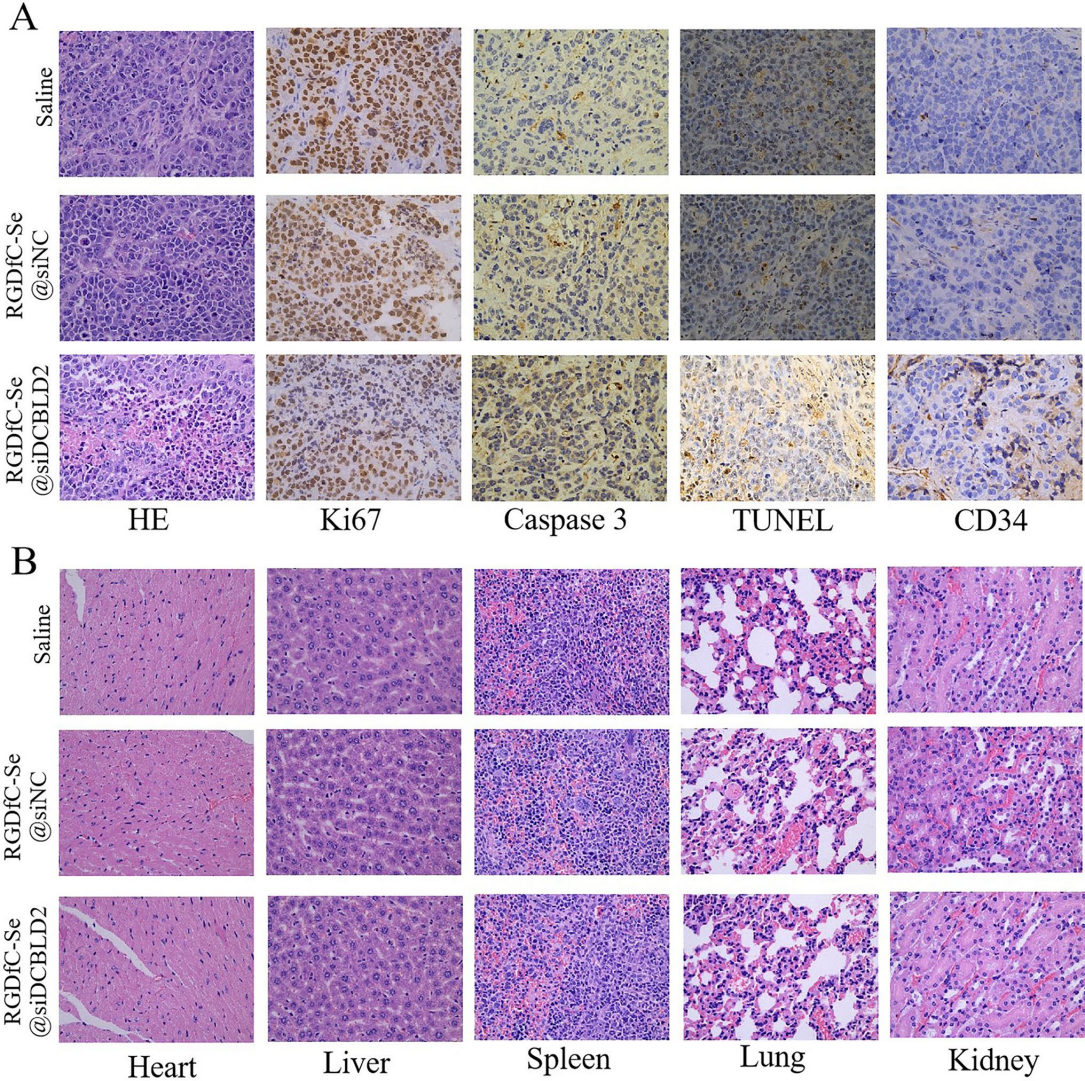
**a** Immunohistochemical analyses of tumor tissues from tumor-bearing mice after treatment with RGDfC-Se@siDCBLD2. **b** H&E analyses of major organs including heart, liver, spleen, lung, and kidney from tumor-bearing mice after treatment with RGDfC-Se@siDCBLD2

Further, we performed hematoxylin–eosin (H&E) staining and immunohistochemical assay to explore the in vivo anti-CRC mechanism of RGDfC-Se@siDCBLD2 in the HCT-116 tumor xenograft model. The tumor tissues were stained with H&E, Ki67 (cell proliferation), Caspase-3 (cell apoptosis), TUNEL (cell apoptosis), and CD34 (angiogenesis). As shown in H&E staining, RGDfC-Se@siDCBLD2 treatment reduced the number of cancerous cells (Fig. 7a, Fig. S7). The Ki67-positive cancer cells in the RGDfC-Se@siDCBLD2-treated group were decreased significantly, indicating that the tumor cell proliferation was remarkably inhibited by RGDfC-Se@siDCBLD2. Moreover, the tumor tissue in RGDfC-Se@siDCBLD2-treated mice exhibited more remarkable caspase-3 (dark brown) and TUNEL signal (brownish yellow) than those from mice treated with saline and RGDfC-SeNPs@siNC, indicating that the RGDfC-Se@siDCBLD2 promoted apoptosis of the tumor cells. After RGDfC-Se@siDCBLD2 treatment, the expression of CD31 (the vascular marker) in tumor cells also reduced obviously, confirming the anti-angiogenesis effect of RGDfC-Se@siDCBLD2. Our results demonstrated that RGDfC-Se@siDCBLD2 exhibited effective anti-CRC effect in vivo by suppressing tumor cell proliferation, lessening vessel formation, and promoting tumor cell apoptosis.

### In vivo biocompatibility of RGDfC-Se@siDCBLD2

The in vivo toxicity of CRC-tarted RGDfC-Se@siDCBLD2 was evaluated in this study by measuring the histopathological changes in the main organs. The H&E staining results show that no histopathology was detected in the major organs, including heart, liver, spleen, lung, and kidneys, of healthy mice after i.v. injection of the RGDfC-Se@siDCBLD2 (Fig. 7b) as compared to the control group, indicating that the RGDfC-Se@siDCBLD2 exhibited good biocompatibility, and could be used as a potential therapeutic drug for CRC therapy.

## Conclusions

In this study, we designed and synthesized functionalized selenium nanoparticles conjugated with the tumor-targeted polypeptide RGDfC, as a gene vector to deliver siDCBLD2 (RGDfC-Se@siDCBLD2) for CRC therapy. With the modification of tumor-targeted polypeptides RGDfC, RGDfC-Se@siDCBLD2 could be effectively internalized by tumor cells mainly through clathrin-mediated endocytosis pathway. In the acidic tumor environment, siDCBLD2 could be efficiently released from the surface of RGDfC-SeNPs. In the in vitro experiments, RGDfC-Se@siDCBLD2 remarkably inhibited the migration of HCT-116 cells and induced tumor cell apoptosis through the down-regulation of DCBLD2 expression. In the in vivo experiments, RGDfC-Se@siDCBLD2 could specially accumulate in the tumor-disease site and suppress the growth of tumor cells with no obvious side effect. RGDfC-modified Se NPs can target tumor to deliver siRNA and achieve good cellular uptake of siRNA, which exerts anti-tumor effect by silencing the expression of specific genes. Based on all these findings, we believed that RGDfC-Se@siDCBLD2 may serve as an innovative chemotherapeutic candidate in the treatment of CRC.

## Supplementary Information

**Additional file 1**.

## Data Availability

All data generated or analysed during this study are included in this published article [and its supplementary information files].

## Abbreviations

CRC: Colorectal cancer
DMEM: Dulbecco’s modified Eagle’s medium
DOG: 2-Deoxy-d-glucose
FBS: Fetal bovine serum
FTIR: Fourier transform infrared spectrometry
HCC: Hepatocellular carcinoma
HCT-116: Human colon cancer cells
MTT: Thiazolylbluetetrazolium bromide
Δψm: Mitochondrial membrane potential
NaN_3_: Sodium azide
NCM460: Human normal colon cells
NPs: Nanoparticles
PBS: Phosphate-buffered saline
RGDfC-Se@siDCBLD2: SiDCBLD2-loaded functionalized selenium nanoparticles modified with RGDfC peptide
Se: Selenium
SeNPs: Se nanoparticles
siRNA: Small interfering RNA
TEM: Transmission electron microscopy
