# Supplementary material for: Engineering siRNA-loaded and RGDfC-targeted selenium nanoparticles for highly efficient silencing of DCBLD2 gene for colorectal cancer treatment
Source: Discov Nano. 2023 Jul 21;18(1):94. doi: 10.1186/s11671-023-03870-0 (PMC10361954; doi:10.1186/s11671-023-03870-0)
Supplement: Supplementary file 1 — Additional file 1. [file 11671_2023_3870_MOESM1_ESM.docx]

Supplementary information for “**Engineering siRNA-loaded and RGDfC-targeted selenium nanoparticles for highly efficient silencing of DCBLD2 gene for colorectal cancer treatment**”

Hongli Huang^1,2,#^, Hanqing Chen ^1, 2,#^, Diwen Shou^1, 2^, Ying Quan^1, 2^, Jiemin Cheng^1, 2^, Huiting Chen^1, 2^, Gang Ning^1, 2^, Yongqiang Li^1, 2^, Yu Xia ^1, 2,^*, Yongjian Zhou ^1, 2,^*

^1^Department of Gastroenterology and Hepatology, Guangzhou First People’s Hospital, School of Medicine, South China University of Technology, Guangzhou,510180, China

**^2^**Guangzhou Key Laboratory of digestive diseases,Guangzhou Digestive Disease Center, Guangzhou First People’s Hospital,Guangzhou 510180, China

**^#^**These authors contributed equally to this work.

* Corresponding authors: [eyyuxia@scut.edu.cn](mailto:eyyuxia@scut.edu.cn) (Yu Xia); [eyzhouyongjian@scut.edu.cn](mailto:eyzhouyongjian@scut.edu.cn) (Yongjian Zhou)

**
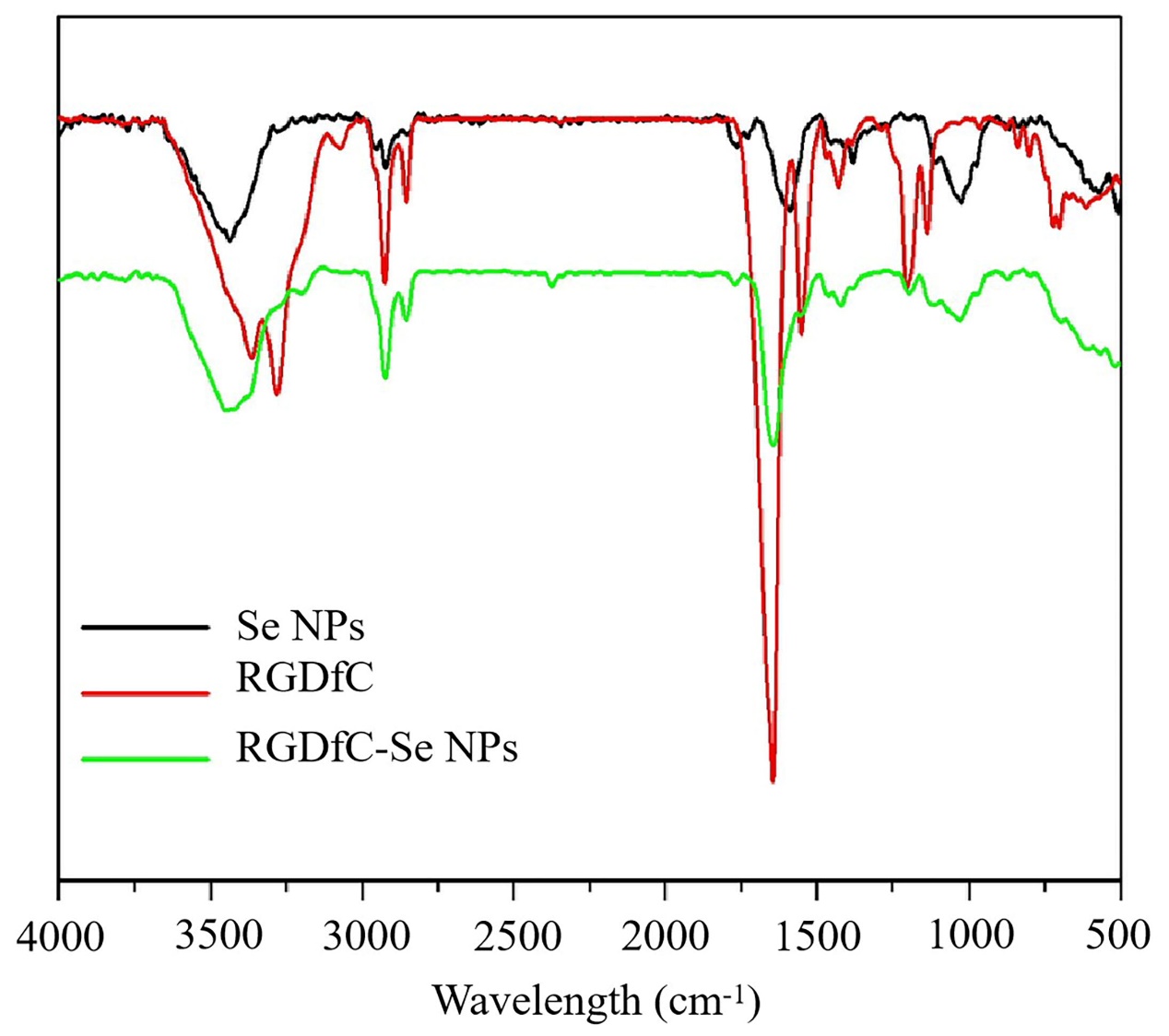
**

**Fig. S1** Fourier transform infrared spectra of SeNPs, RGDfC and RGDfC-SeNPs.

**
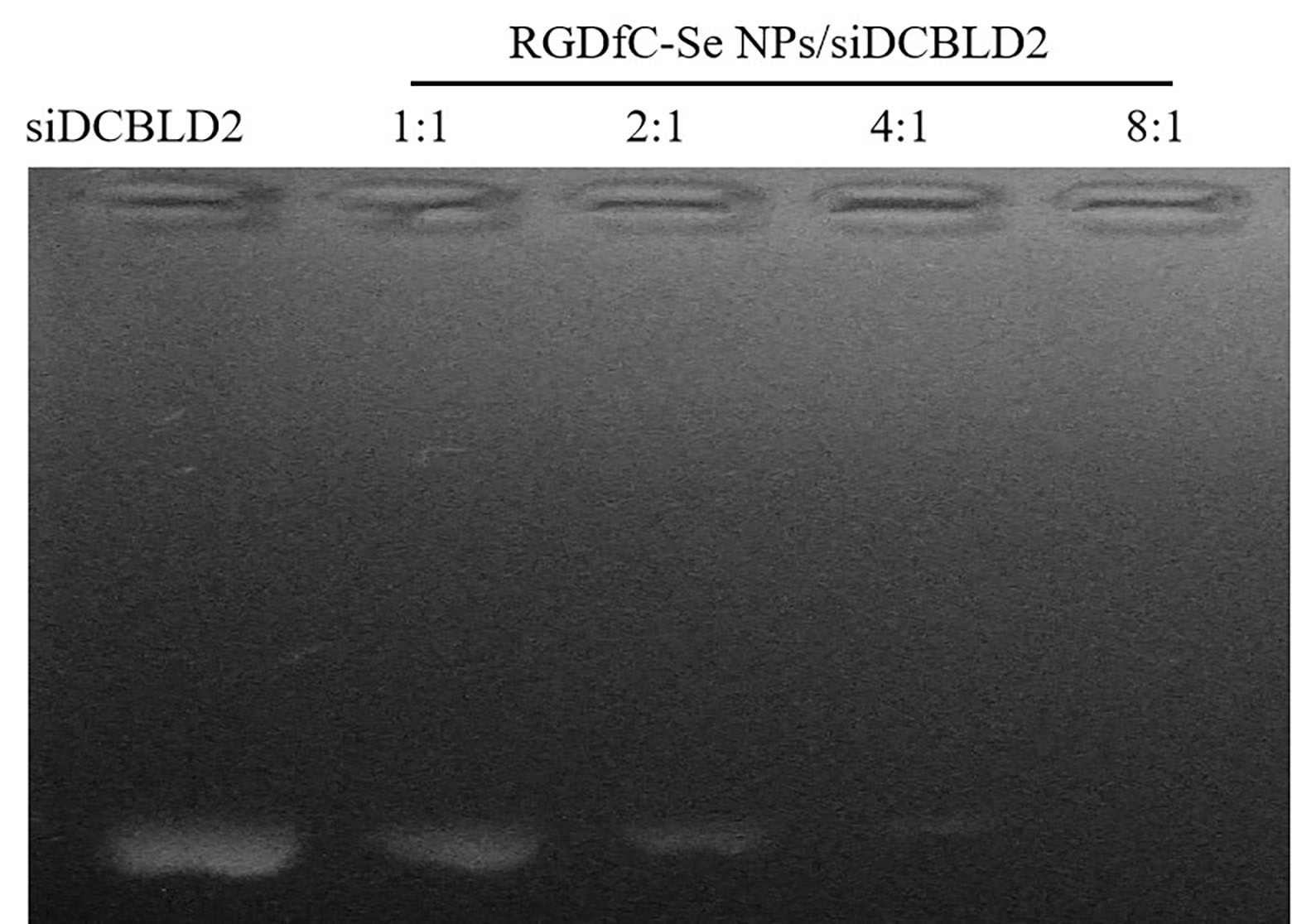
**

**Fig. S2** The stability of siDCBLD2 loaded onto RGDfC-SeNPs was investigated by agarose gel electrophoresis, and the weight ratio of RGDfC-SeNPs/siDCBLD2 was from 1/1 to 8/1.


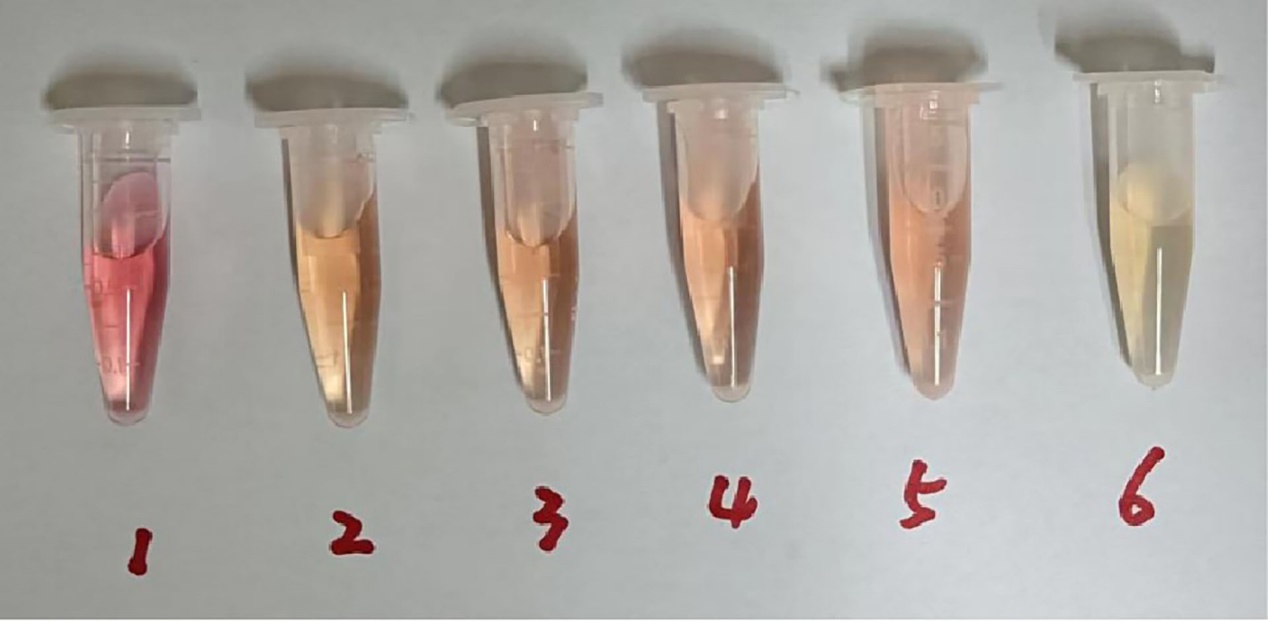


**Fig. S3** The siRNA stability analysis using serum protein containing media. 1 indicates media; 2 indicates serum protein containing media; 3 indicates the mixed solutions including the serum protein containing media and RGDfC-Se@DCBLD2 (50 nM); 4 indicates the mixed solutions including the serum protein containing media and RGDfC-Se@DCBLD2 (100 nM); 5 indicates the mixed solutions including the serum protein containing media and RGDfC-Se@DCBLD2 (200 nM); 6 indicates the RGDfC-Se@DCBLD2 solution (200 nM).


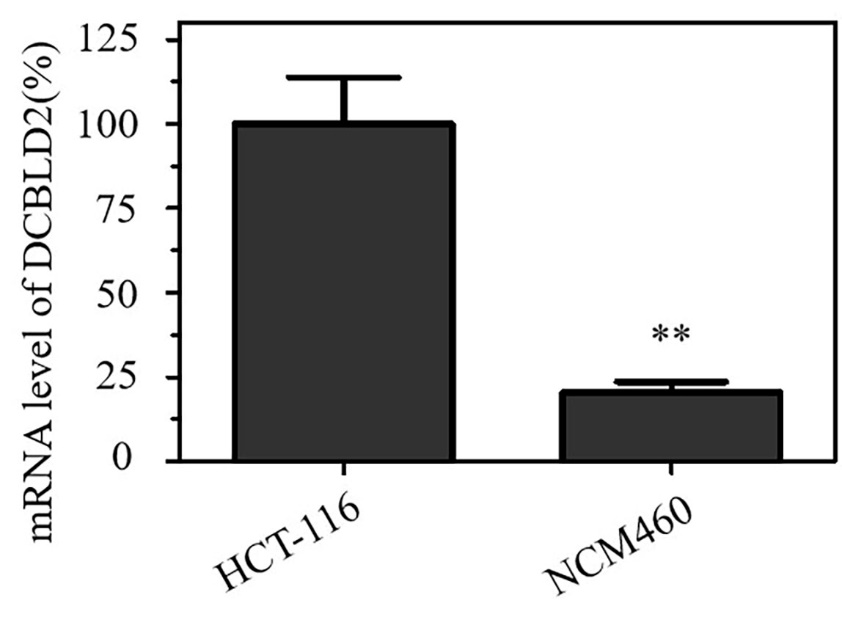


**Fig. S4** The mRNA level of DCBLD2 in HCT-116 cells and NCM460 cells. ***p*<0.01 *vs* HCT-116 cells.


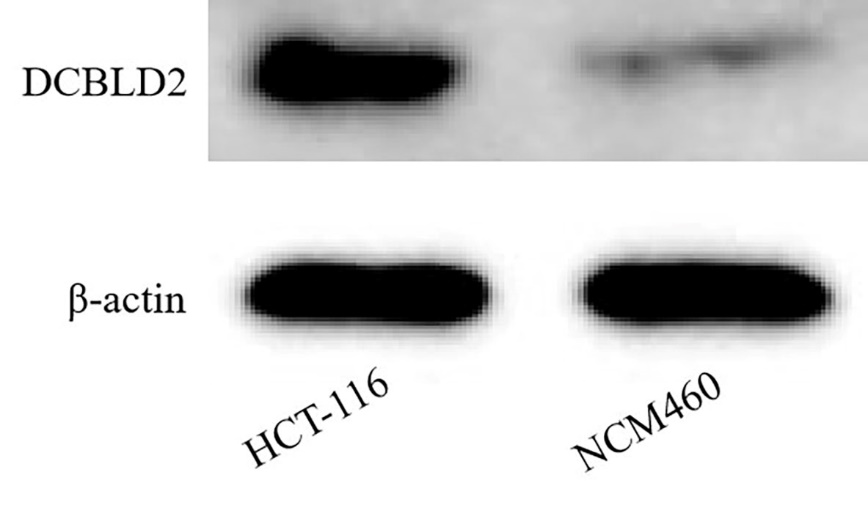


**Fig. S5** The protein level of DCBLD2 in HCT-116 cells and NCM460 cells.


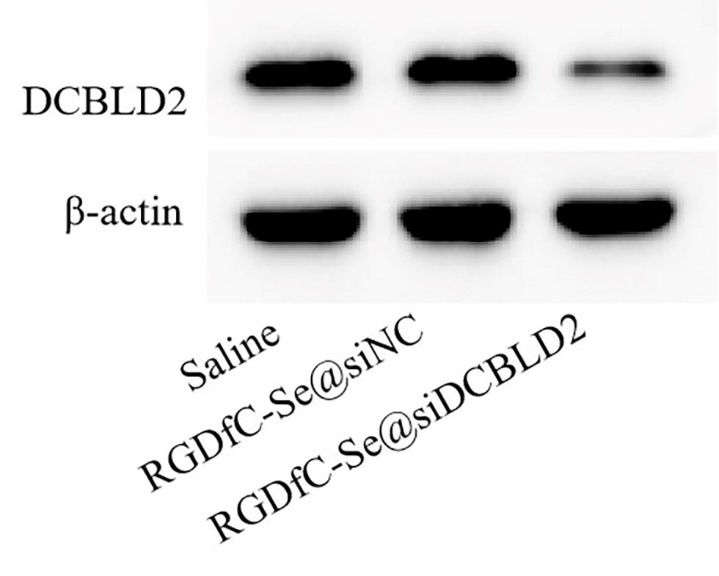


**Fig. S6** The protein expressions of DCBLD2 in the tumors treated with saline, RGDfC-Se@siNC and RGDfC-Se@siDCBLD2, respectively.


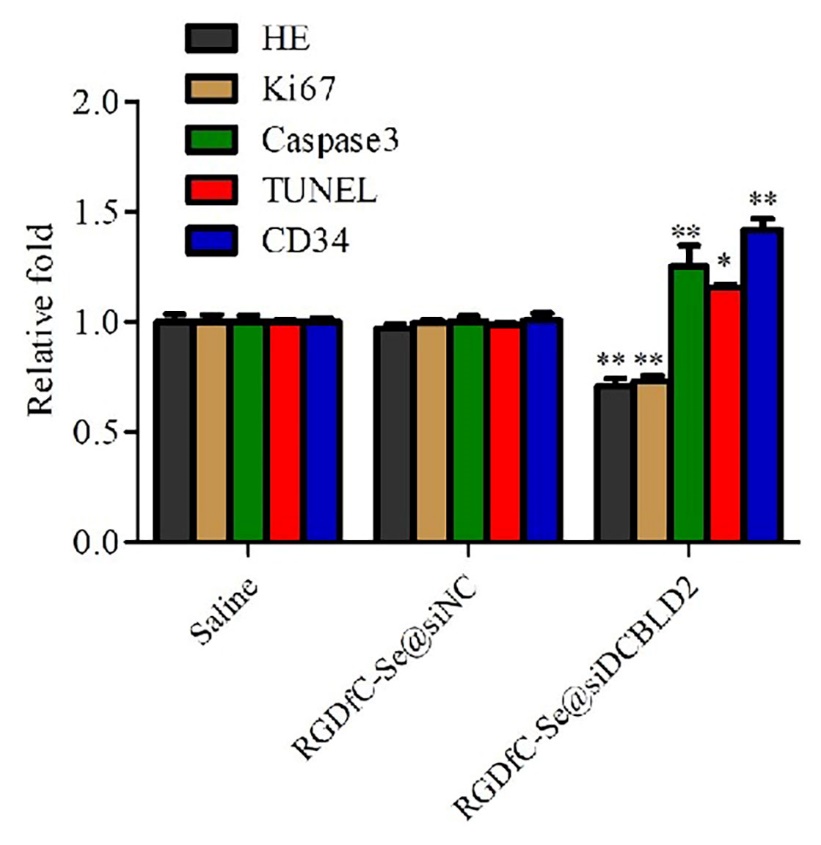


**Fig. S7** The quantitative analysis of immunohistochemical assays from Fig. 7A. **p*<0.05, ***p*<0.01 *vs* saline group.

**The uncropped images of gels and Western blots**


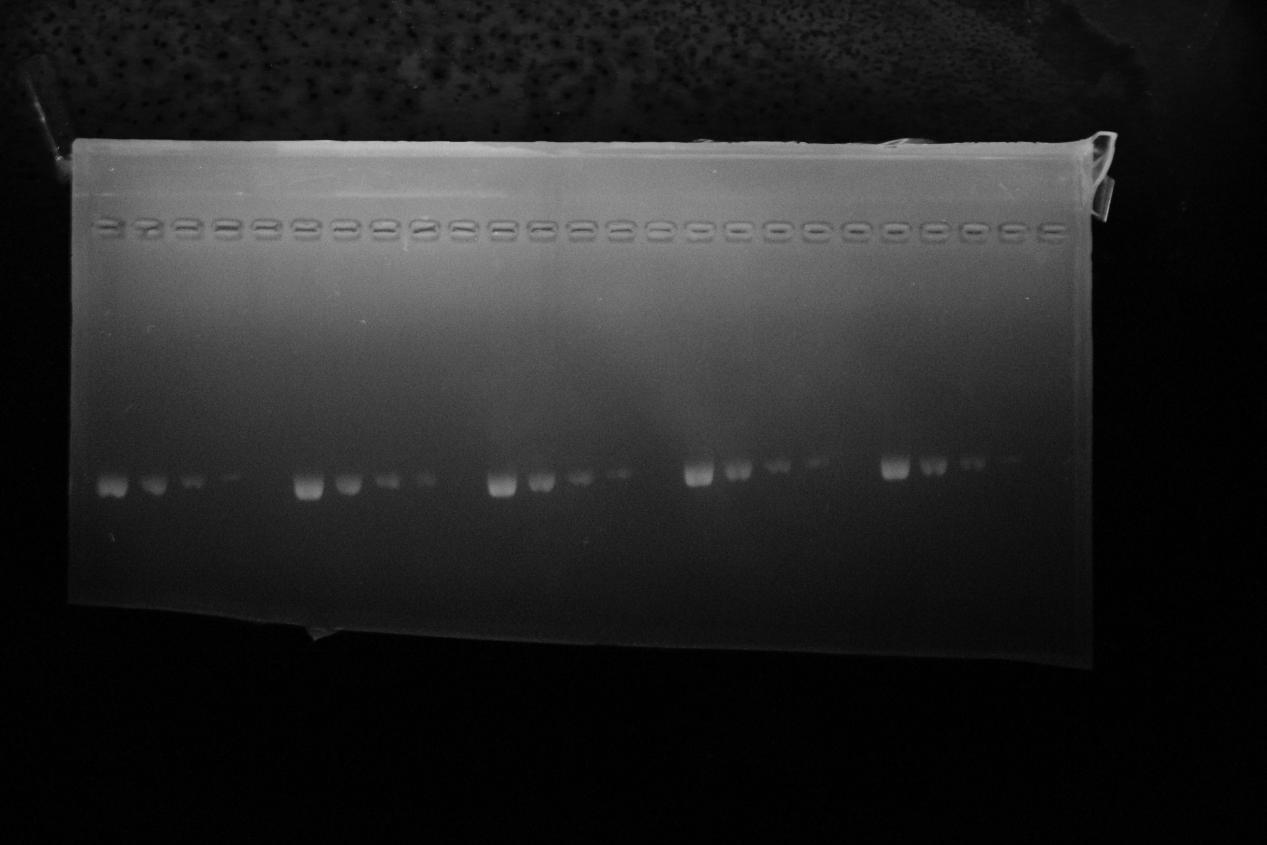


**Fig. S2** The uncropped gel image (lane1-5).


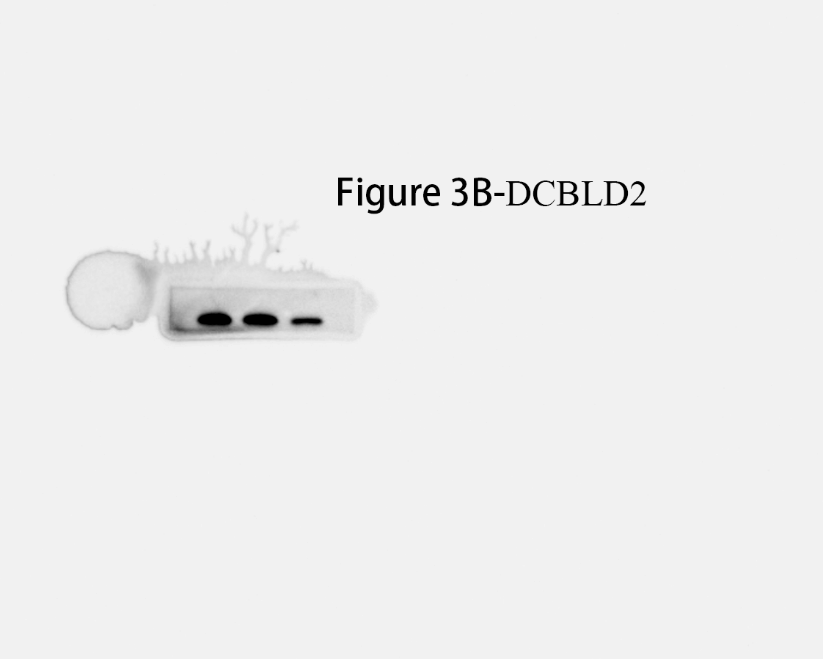


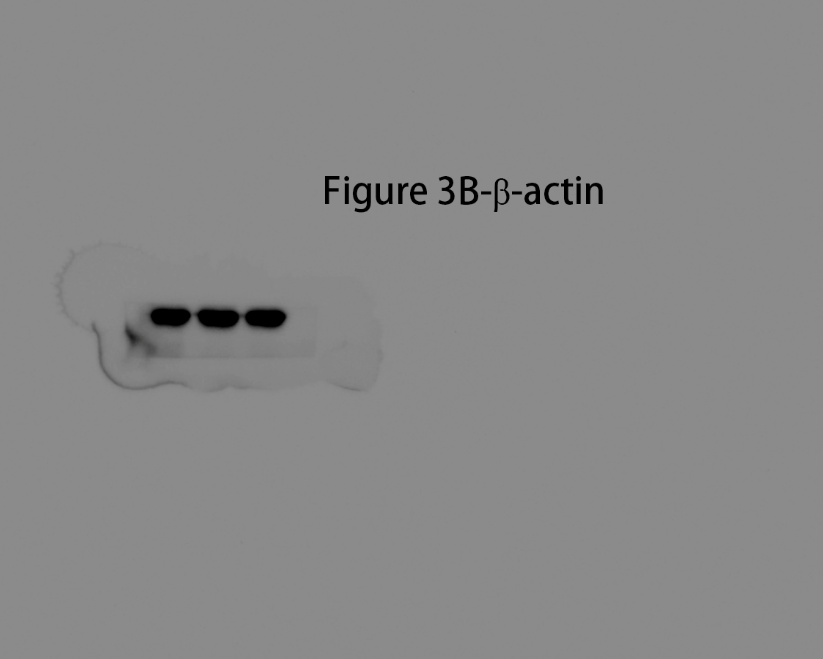


**Fig. 3B** The uncropped WB image.


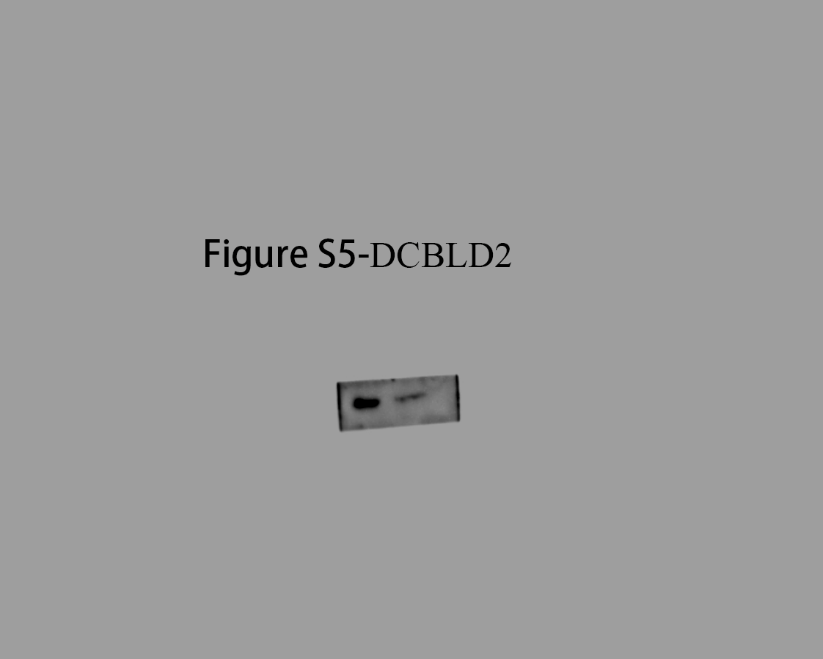


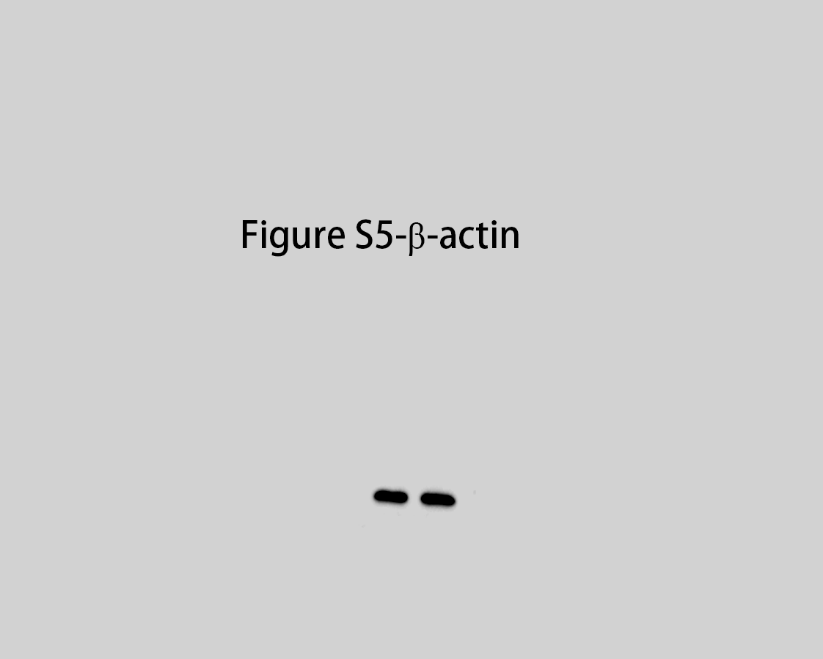


**Fig. S5** The uncropped WB image.


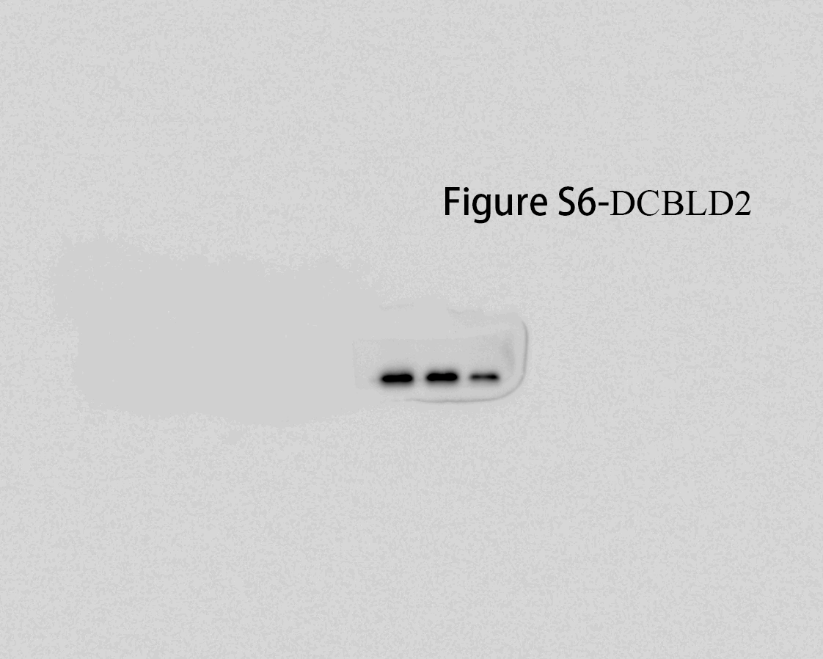


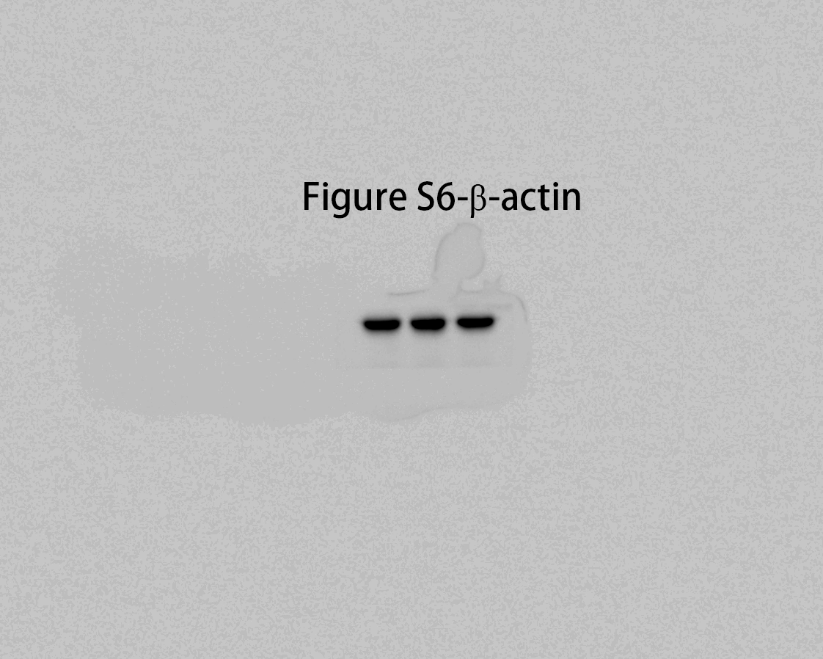


**Fig. S6** The uncropped WB image.
